# Engineered proteins to restore and enhance neurotransmitter release

**DOI:** 10.64898/2026.10.23.761409

**Published:** 2026-10-24

**Authors:** Morven Chin, Ke Yang, Ricardo C. López, Chao Tan, Pascal S. Kaeser

## Abstract

Synapse dysfunction is a hallmark feature of many neurological diseases. Synaptic transmission is initiated in nerve terminals, where active zone protein complexes control vesicle fusion for neurotransmitter release. Here, we engineer proteins that mediate active zone functions and demonstrate their use for reconstituting and strengthening neurotransmission. A protein composed of Ca _V_ β4 and the RIM1 zinc finger, β4-Zn, restored neurotransmitter release after active zone disruption through recruitment of Munc13. We develop β4-MC, derived from the Munc13-1 MUN and C2C domains, which bypasses Munc13 recruitment and directly mediates vesicle docking, priming and fusion after active zone disruption. In β4-MC, MUN domain functions are dispensable for docking, but active MUN and C2C domains are both necessary for neurotransmitter release. In wild-type mouse and human neurons, β4-MC but not β4-Zn strengthens neurotransmitter release. Furthermore, β4-Zn and β4-MC restore and enhance dopamine release in the mouse striatum, respectively. Hence, β4 proteins are engineered secretory proteins with therapeutic potential as enhancers of synaptic function.

## Full Text Availability

The license terms selected by the author(s) for this preprint version do not permit archiving in PMC. The full text is available from the preprint server.

